# Symptomatic Mullerian Duct Cyst in a Male Infant

**DOI:** 10.21699/ajcr.v7i4.449

**Published:** 2016-09-01

**Authors:** Abhishek Chinya, Prince Raj, Shandip Kumar Sinha, Yogesh Kumar Sarin

**Affiliations:** Department of Pediatric Surgery, Maulana Azad Medical College, New Delhi, India

**Keywords:** Mullerian duct cyst, Intestinal obstruction, Obstructive uropathy

## Abstract

Symptomatic Mullerian duct cyst is a rare entity in children. A 9-month-old male infant presented with bowel and urinary obstructive symptoms. Imaging investigations revealed a cystic mass in the rectovesical pouch compressing bladder neck and rectum. At laparotomy, a Mullerian duct cyst was found. Most of the cyst was excised and the remaining cyst mucosa was cauterized. The child improved thereafter.

## CASE REPORT

A 9-month-old male infant presented with urinary retention. The parents also gave history of constipation and passing of small amount of urine per void since the last three months. Abdominal examination revealed distended bladder. Child was catheterized and enema was given for rectal evacuation. Following bladder decompression a smooth, firm lump of approximately 7 cm X 5 cm was palpable in the hypogastrium. Rectal examination revealed a firm lump which was palpable anteriorly. Ultrasound showed an anechoic cystic lesion posterior to the urinary bladder with septations within it. CECT abdomen revealed a 7 cm X 3 cm X 2.5 cm well defined elongated fluid attenuating hypodense cyst in the recto-vesical pouch. There were no calcifications within the mass. The mass was causing compression on the bladder anteriorly and the rectum and sigmoid colon posteriorly. The urinary bladder showed diffuse wall thickening.

The child was taken up for cystoscopy and laparoscopic cyst excision. Cystoscopy did not show any communication of the cyst with the posterior urethra. The bladder was found to be trabeculated. Laparoscopy showed hypertrophied bladder and thick walled cystic swelling posterior to bladder and anterior to rectum compressing both.[Fig. 1 and 2] The cyst was densely adhered to rectum and bladder. Adhesiolysis was difficult laparoscopically and hence open deroofing of the cyst was done. Both vas deferens were identified and saved. Majority of the cyst was excised and the remaining cyst cavity mucosa was cauterized. A drain was placed in rectovesical space. Postoperatively, the child improved and was discharged. The fluid cytology revealed few inflammatory cells in a proteinous background. Histopathological analysis of the excised wall showed largely ulcerated areas with focal areas showing intact mucosa formed by cuboidal epithelium consistent with the diagnosis of Mullerian duct cyst. The child presented for follow up in outpatient department after three months and was doing well without any symptoms.

**Figure:**
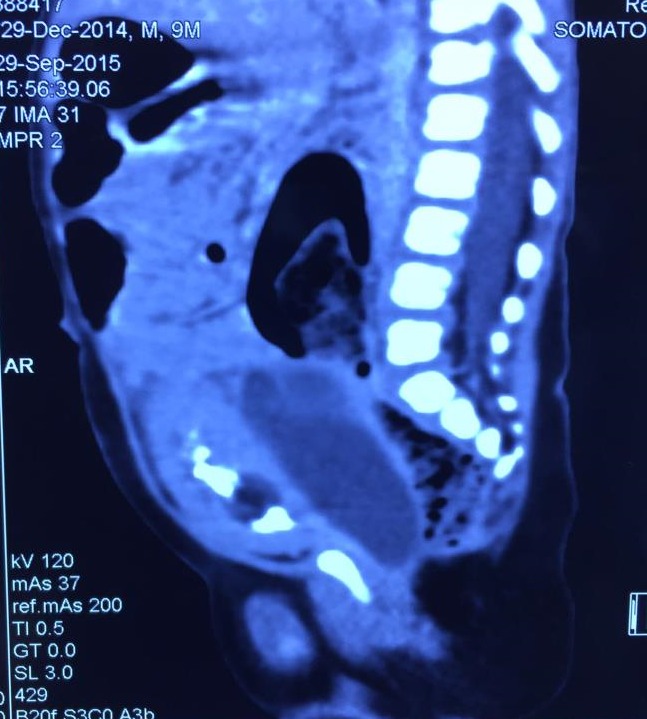
Figure 1: CECT showing cystic mass posterior to bladder and anterior to the rectum.

**Figure:**
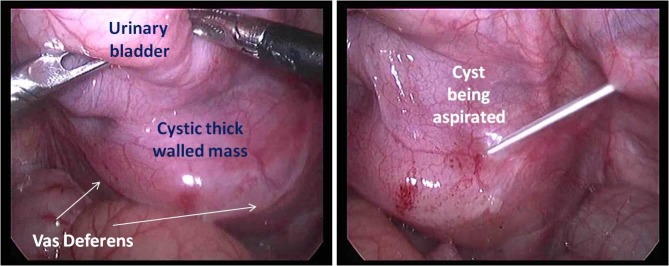
Figure 2: Laparoscopic view of cyst and its relation to surrounding structures.

## DISCUSSION

Anti Mullerian Hormone in males leads to regression of the paramesonephric duct. Failure of regression and focal dilatation leads to the development of Mullerian duct cysts. The differential diagnosis of midline pelvic swellings in the male include prostatic utricle cyst, prostatic retention cysts, prostatic abscesses, bladder diverticulum, rectal duplication cyst or any pelvic neoplasm. Trans-rectal ultrasound, CT and magnetic resonance imaging are useful in diagnosis.[1,2] Prostatic utricle cysts generally present within the first two decades and may communicate with the posterior urethra resulting in post void dribbling. They also do not extend beyond the base of the prostate and are associated with hypospadias and disorders of sexual differentiation.[3] Such a large pelvic cyst extending beyond the prostate with no communication with the urethra led us to make a diagnosis of Mullerian duct cyst.

Mullerian duct cyst may present with urinary retention, urinary tract symptoms, renal failure or constipation as was the scenario in the index case. [4-7] All these cases were mostly reported from adult population except the one from which was in 5 year old child.[7] There are also reports of association of such cysts with renal agenesis and malignant transformation.[8,9] Rarely it may present in infancy with mass effect leading to urinary and bowel obstructive symptoms as found in the index case.

Treatment of small asymptomatic cysts remains conservative. Transurethral deroofing of cyst and laparoscopic excision are the options for small cyst but for large pelvic cyst, as in our case, surgical excision is a better option. Robot- assisted laparoscopic excision has also been reported in boys and young adults.[10]

To summarize, Mullerian duct cysts though rare anomaly, should be kept in the differential diagnosis of midline swellings in rectovesical cystic location. Transrectal ultrasound and CT scans are helpful in diagnosis. Surgical excision of the cyst results in good recovery.

## Footnotes

**Source of Support:** Nil

**Conflict of Interest:** None declared

